# Development and Validation of the Anxious Distress Assessment Scale (ADS) in Patients with Major Depressive Disorder

**DOI:** 10.3390/healthcare14070880

**Published:** 2026-03-30

**Authors:** Ai Hwa Lim, Jesjeet Singh Gill, Chong Guan Ng

**Affiliations:** 1 Department of Psychological Medicine, Faculty of Medicine, Universiti Malaya, Kuala Lumpur 50603, Malaysia; jesjeet@gmail.com; 2 Malaysian Psychiatric Association, Petaling Jaya 46300, Malaysia; chong.guan.um@gmail.com

**Keywords:** anxious distress, self-report, mood disorders, validation

## Abstract

**Objective**: Anxiety symptoms frequently occur alongside mood disorders and are associated with poorer clinical outcomes, highlighting the importance of early and accurate detection. This study evaluated the diagnostic accuracy and psychometric properties of the Anxious Distress Assessment Scale (ADS), a newly developed brief self-report instrument designed to detect anxious distress. **Method**: The study was conducted in two phases. Phase 1 involved the development of the ADS as a five-item instrument reflecting the DSM-5-TR anxious distress criteria. In Phase 2, 105 adults diagnosed with major depressive disorder (MDD) completed the ADS alongside the Generalized Anxiety Disorder-7 (GAD-7) and the Montgomery–Åsberg Depression Rating Scale (MADRS). Psychometric evaluation included internal consistency reliability (Cronbach’s α), analyses of convergent validity, and diagnostic accuracy assessment using correlation and receiver operating characteristic (ROC) analyses. **Results**: Anxious distress was highly prevalent, with 71% of participants meeting DSM-5-TR criteria. The ADS demonstrated strong diagnostic performance, with sensitivity of 88.0%, specificity of 90.0%, positive predictive value of 95.7%, and negative predictive value of 75.0%. ROC analysis yielded an area under the curve (AUC) of 0.97 (95% CI: 0.943–0.997), with an optimal cut-off score of ≥10. Internal consistency was excellent (Cronbach’s α = 0.897). Principal component analysis supported a unidimensional structure, accounting for 71.5% of the total variance, with all items loading above 0.80. The ADS also demonstrated strong convergent validity, correlating significantly with the GAD-7 (r = 0.82) and MADRS (r = 0.68). **Conclusions**: The ADS demonstrates promising psychometric properties, including strong reliability, meaningful convergent validity, and excellent diagnostic accuracy. Its brief format and direct alignment with DSM-5-TR anxious distress criteria support its potential utility as a practical screening tool in clinical settings. However, these findings should be interpreted in light of the study’s focus on English-speaking Malaysian adults with MDD recruited from a tertiary-care setting. Further validation across diagnostic groups, clinical contexts, and cultural and linguistic populations is warranted.

## 1. Introduction

Mood disorders encompass a wide range of emotional, cognitive, and behavioral symptoms. This variability led DSM-5 work groups to introduce several specifiers that allow clinicians to identify clinically meaningful subgroups, including melancholic features, mixed features, and anxious distress [1]. Among these, the anxious distress specifier has received increasing attention. It is frequently observed in both depressive and bipolar disorders and has been consistently associated with more complex illness trajectories, including higher relapse rates, longer episode duration, and increased risk of suicidal behavior [1,2,3,4]. In addition, anxious distress may influence treatment outcomes, as individuals meeting criteria for this specifier often respond differently to standard antidepressant treatments compared with those without it. Although other specifiers, such as mixed features, also play important roles in characterizing mood disorder subtypes, the anxious distress specifier is particularly notable because of its broad clinical relevance and strong prognostic value. Consequently, it has become an important focus in both clinical practice and research on mood disorders.

Accurate identification of anxious distress can help clinicians recognize patients who may benefit from more individualized treatment approaches and closer clinical monitoring. According to the DSM-5-TR, the anxious distress specifier is applied when at least two of the following symptoms are present on the majority of days during a major depressive episode [1]:

- Feeling keyed up or tense;
- Feeling unusually restless;
- Difficulty concentrating due to worry;
- Fear that something awful may happen;
- Fear that one might lose control of oneself.

The severity of anxious distress is determined by the number of symptoms present [1]:

- 2 symptoms: Mild;
- 3 symptoms: Moderate;
- 4–5 symptoms: Moderate to severe;
- 4–5 symptoms with psychomotor agitation: Severe.

Measurement-based practice has become an important component of contemporary mental health care, supporting systematic monitoring of symptom changes and evidence-based treatment decisions [5]. Within this framework, standardized instruments are essential for reliably assessing symptom severity and tracking clinical progress. A brief and accurate measure of anxious distress may therefore facilitate early identification of high-risk patients and improve the monitoring of treatment outcomes.

One instrument developed to operationalize the DSM-5 anxious distress specifier is the DSM-5 Anxious Distress Specifier Interview (DADSI), created by Zimmerman et al. [6]. This clinician-administered interview assesses anxious distress symptoms during the previous week while also determining whether they have been present throughout the depressive episode. In the Rhode Island MIDAS Project, 78% of 173 patients with depression met criteria for anxious distress using the DADSI, which demonstrated strong reliability, validity, and sensitivity to symptom change [5,6]. A key advantage of the DADSI is its structured clinician-guided format, which allows clarification of ambiguous responses and may reduce diagnostic misclassification. However, accurate scoring requires substantial clinical judgment, as the rating categories can involve subtle distinctions. For example, the scoring categories for “feeling keyed up or tense” range from “not at all” to “very tense most of the time,” with intermediate categories requiring nuanced clinical interpretation. These distinctions may be challenging for less experienced clinicians and may limit the instrument’s feasibility in routine clinical practice.

In addition to clinician-administered approach, Zimmerman et al. developed a self-report format, the Clinically Useful Depression Outcome Scale Anxious Distress Subscale (CUDOS-A), which incorporates the five anxious distress symptoms within the broader Clinically Useful Depression Outcome Scale (CUDOS) [7]. The CUDOS-A demonstrated good psychometric properties, including satisfactory internal consistency (α = 0.79) and strong test–retest reliability (r = 0.89) [7]. However, because the CUDOS-A is embedded within a broader depression severity instrument, it may be less suitable when anxious distress needs to be assessed independently. Moreover, although DSM-5-TR specifies that anxious distress may occur in both major depressive disorder and bipolar disorder, validation studies of the CUDOS-A have largely focused on individuals with unipolar depression [1,7,8]. Its integration within a longer measure may also limit its practicality in time-constrained clinical environments.

These limitations highlight the need for a concise, stand-alone self-report instrument that directly operationalizes DSM-5-TR anxious distress criteria. Such a measure would allow clearer conceptual focus, more flexible use across different mood disorder populations, and faster administration in both clinical and research settings.

To address this need, the present study aimed to develop and validate the Anxious Distress Assessment Scale (ADS), a brief self-report instrument grounded in DSM-5-TR anxious distress criteria. The objective was to create a practical tool suitable for screening and monitoring anxious distress among individuals with major depressive disorder, while supporting timely clinical decision-making and measurement-based care across diverse healthcare settings.

## 2. Materials and Methods

The study was carried out in two sequential phases.

### 2.1. Phase 1: Scale Development

Phase 1, conducted from May to July 2024, involved the development of the Anxious Distress Assessment Scale (ADS), an English-language instrument designed to reflect the DSM-5-TR definition of anxious distress [1]. The scale comprises five items, each intentionally constructed to correspond directly to one of the five DSM-5-TR symptoms defining the anxious distress specifier (Table 1).

The initial draft of the ADS was reviewed by two consultant psychiatrists with more than 20 years of clinical experience in managing mood and anxiety disorders. The experts were selected based on their extensive experience in diagnosing and treating depressive and anxiety disorders and their familiarity with DSM-5-TR diagnostic criteria. Each reviewer independently evaluated the items for conceptual relevance to DSM-5-TR anxious distress symptoms, clarity of wording, appropriateness of response anchors, and clinical interpretability. Based on expert feedback, the items were considered conceptually aligned with DSM-5-TR criteria and clearly worded for patient self-report, and no substantive modifications were required beyond minor stylistic adjustments to improve readability.

Although quantitative indices such as the Content Validity Index (CVI) may be used to summarize expert ratings, COSMIN guidance indicates that content validity can also be established through qualitative evaluation involving both experts and members of the target population during early instrument development [9]. Accordingly, the present study emphasized expert clinical review together with participant feedback to ensure conceptual alignment with DSM-5-TR anxious distress criteria and comprehensibility for intended respondents.

During the early phase of data collection, an embedded pilot-testing process was conducted in which participants were informally asked whether any items were unclear or difficult to interpret after completing the ADS. Feedback indicated that the items were generally straightforward and understandable, and no substantial comprehension difficulties were reported. Although formal cognitive interviewing procedures were not conducted, this feedback provided preliminary confirmation of item clarity within the target population.

The ADS consists of five items rated on a 0–4 Likert scale, yielding a total score ranging from 0 to 20, with higher scores indicating greater severity of anxious distress symptoms. Total scores were used for all analyses; individual items were not dichotomized, as the ADS was designed to function as a dimensional screening measure rather than a categorical diagnostic tool.

Each ADS item is scored on a 0–4 Likert scale:

0 = Never;

1 = Rarely (less than 1 day/week);

2 = Sometimes (1–2 days/week);

3 = Frequent (3–4 days/week);

4 = A great deal of the time (almost every day).

Participants were instructed to rate each item with reference to their current depressive episode, focusing on symptom frequency during the recent period of that episode rather than a fixed recall window (e.g., the past week). The phrase “During your current episode of depression” was presented as a shared stem preceding all five ADS items to maintain conceptual alignment with the DSM-5-TR anxious distress specifier, which defines symptoms as occurring on the majority of days during the depressive episode rather than within a fixed retrospective timeframe.

This approach aligns with COSMIN guidance that recall periods should be conceptually appropriate to the construct being measured and feasible for patient self-report when diagnostic criteria do not specify a standardized timeframe [9]. The questions were as follows:

During your current episode of depression:

1. Have you been feeling nervous, tense, or unable to relax most of the time (feeling keyed up or tense)?
2. Have you been feeling extremely restless such that you were unable to sit still most of the time (feeling unusually restless)?
3. Have you been having difficulty concentrating due to worry or anxiety most of the time (difficulty concentrating due to worry)?
4. Have you been worrying that something bad will happen most of the time (fear that something awful might happen)?
5. Have you been worrying that you may lose control of yourself most of the time (fear that one might lose control)?

### 2.2. Phase 2: Participant Recruitment

Phase 2 was carried out over a ten-month period, from August 2024 to May 2025, at the University of Malaya Medical Centre (UMMC), a tertiary academic hospital providing both inpatient and outpatient psychiatric services. Participants were recruited from both the psychiatric ward and outpatient clinic. The research protocol received ethical approval from the relevant institutional review board, and written informed consent was obtained from all participants prior to enrolment.

The sample included 105 patients with a verified diagnosis of Major Depressive Disorder (MDD), recruited using convenience sampling. Eligible participants were aged 18–65 years, had an established clinical diagnosis of MDD, possessed sufficient English proficiency to complete the study measures, and were capable of providing informed consent. Individuals were excluded if they were medically unstable, experiencing delirium, displaying active psychotic symptoms, or presenting with significant cognitive difficulties due to neurological or medical conditions.

Sample size determination followed established psychometric recommendations. Standard guidance for factor analysis suggests subject-to-item ratios ranging from 3:1 to 20:1, and Gorsuch (1983) recommends a minimum of five participants per item with at least 100 total participants regardless of scale length [10]. With five ADS items, a sample size of *N* = 105 exceeded the minimum requirements for factor-analytic and reliability analyses. Participation was voluntary, and no financial or material compensation was provided.

### 2.3. Clinical Assessment

All participants underwent a comprehensive psychiatric evaluation conducted by a psychiatry medical officer with eight years of clinical experience in mood and anxiety disorders. Diagnoses of MDD and the anxious distress specifier were determined through a clinical interview guided by DSM-5-TR criteria.

The “majority of days” criterion was operationalized through clinical evaluation of symptom persistence across the depressive episode, based on patient report and clinician judgment regarding whether symptoms were present for more than half of the episode duration. Clinical judgment guided by DSM-5-TR criteria was used as the reference standard because no widely used structured diagnostic interview currently operationalizes the anxious distress specifier [11,12]. Participants subsequently completed the ADS following the diagnostic interview to minimize expectancy bias. Participants were not informed of their diagnostic classification prior to completing the ADS, and no feedback regarding anxious distress status was provided during assessment. Additional sociodemographic and clinical data were collected, including age, sex, ethnicity, education level, employment status, prior psychiatric hospitalization, previous treatments, substance use, and comorbid psychiatric disorders. When available, supplementary information was obtained from caregivers, family members, or medical records to support the accuracy of patient-reported clinical history.

### 2.4. Comparator Measure

All participants completed the ADS alongside two established comparison instruments: the Montgomery–Åsberg Depression Rating Scale (MADRS) and the Generalized Anxiety Disorder-7 (GAD-7). The MADRS is a clinician-administered instrument widely used to assess depressive symptom severity. It consists of ten items rated on a 0–6 scale, yielding total scores ranging from 0 to 60, with established cut-offs indicating mild, moderate, and severe depression [13]. The MADRS was administered according to standard administration guidelines and was included to evaluate convergent validity, given that anxious distress is conceptualized within depressive episodes [14,15].

The GAD-7 is a concise self-report measure originally developed to screen for generalized anxiety disorder but is now widely used to assess general anxiety symptoms across diverse clinical settings [16]. Because several DSM-5-TR anxious distress features overlap with the symptom domains assessed by the GAD-7, it was included as a comparator measure for convergent validity.

### 2.5. Statistical Analysis

Psychometric evaluation of the ADS was undertaken. Concurrent validity was assessed by comparing ADS scores with DSM-5-TR-based clinical determinations of anxious distress. Diagnostic accuracy was evaluated using sensitivity, specificity, predictive values, likelihood ratios, and receiver operating characteristic (ROC) curve analyses. Receiver Operating Characteristic (ROC) curve analysis was performed to evaluate the diagnostic performance of the ADS. The area under the curve (AUC) and its 95% confidence interval were estimated using the nonparametric method implemented in SPSS. Sensitivity, specificity, and predictive values were calculated from 2 × 2 contingency tables, and 95% confidence intervals were estimated using binomial methods based on the distribution of proportions. Candidate cut-off scores were evaluated using sensitivity, specificity, and the corresponding Youden Index to determine the optimal threshold. Convergent validity was examined through correlations between ADS scores and measures of anxiety and depressive severity. Internal consistency reliability was assessed using Cronbach’s alpha. All statistical analyses were conducted using IBM SPSS Statistics Version 29. A two-tailed significance level of α = 0.05 was used for inferential statistical tests. Descriptive statistics, including means and standard deviations, were calculated for all scale scores. Principal Component Analysis (PCA) was conducted to explore the dimensional structure of the ADS, as it is a newly developed self-report instrument with no previously established factor structure. PCA was selected as an initial data reduction technique to identify the dominant component structure underlying the items. Sampling adequacy was evaluated using the Kaiser–Meyer–Olkin (KMO) measure and Bartlett’s test of sphericity. Components were extracted using PCA, and component retention was guided by eigenvalues greater than 1.0 and inspection of the scree plot.

## 3. Results

### 3.1. Participant Demographics and Clinical Characteristics

Participant demographic and clinical characteristics are summarized in Table 2. The study sample comprised 105 participants, with a mean age of 33.10 years (SD = 10.89). Most participants were female (81.0%, *n* = 85), while males represented 19.0% (*n* = 20). In terms of ethnicity, most participants identified as Malay (66.7%, *n* = 70), followed by Chinese (27.6%, *n* = 29), Indian (4.8%, *n* = 5), and one individual categorized as “other” (1.0%, *n* = 1). Regarding marital status, most were single (66.7%, *n* = 70), with 27.6% (*n* = 29) married, 4.8% (*n* = 5) divorced, and 1.0% (*n* = 1) widowed. Educational attainment also varied across the sample: the largest proportion had tertiary-level education (62.9%, *n* = 66), followed by secondary education (25.7%, *n* = 27), postgraduate qualifications (10.5%, *n* = 11), and one participant (1.0%, *n* = 1) whose highest education level was primary school. All participants completed the study instruments in full, and no missing data were observed for the ADS, MADRS, or GAD-7 measures.

About 71% of participants met the DSM-5-TR criteria for anxious distress. Among the 105 individuals diagnosed with major depressive disorder, 12 were also found to have at least one additional psychiatric condition. The comorbid diagnoses observed included borderline personality disorder, attention-deficit/hyperactivity disorder (ADHD), autism spectrum disorder, substance use disorder, social anxiety disorder, and body dysmorphic disorder. Among participants with comorbid substance use disorder (*n* = 3), the substances involved included alcohol (*n* = 1), cannabis (*n* = 1) and benzodiazepine (*n* = 1). Of the 105 participants, 61 (58.1%) were recruited from outpatient psychiatric services, while 44 (41.9%) were inpatients. Based on MADRS scores at the time of assessment, 7 participants (6.7%) had minimal or no current depressive symptoms, 20 (19.0%) had mild depression, 43 (41.0%) had moderate depression, and 35 (33.3%) had severe depression. All participants received pharmacotherapy during the study period.

### 3.2. Descriptive Statistics

Descriptive analyses were computed for each item of the ADS to assess the central tendency and the variability across the measure. Mean scores for the individual items ranged from 2.00 to 2.59. Item 4 (“Felt that something awful might happen”) showed the highest mean score at 2.59 (SD = 1.45), indicating that this symptom was endorsed most frequently by participants. In contrast, Item 2 (“Felt unusually restless”) had the lowest mean of 2.00 (SD = 1.37), suggesting it was the least commonly reported symptom. The ADS total score had a mean value of 11.94 (SD = 5.69), which reflects a moderate overall level of anxious distress in the sample (Table 3).

### 3.3. Area Under the Curve (AUC)

**Figure 1:**
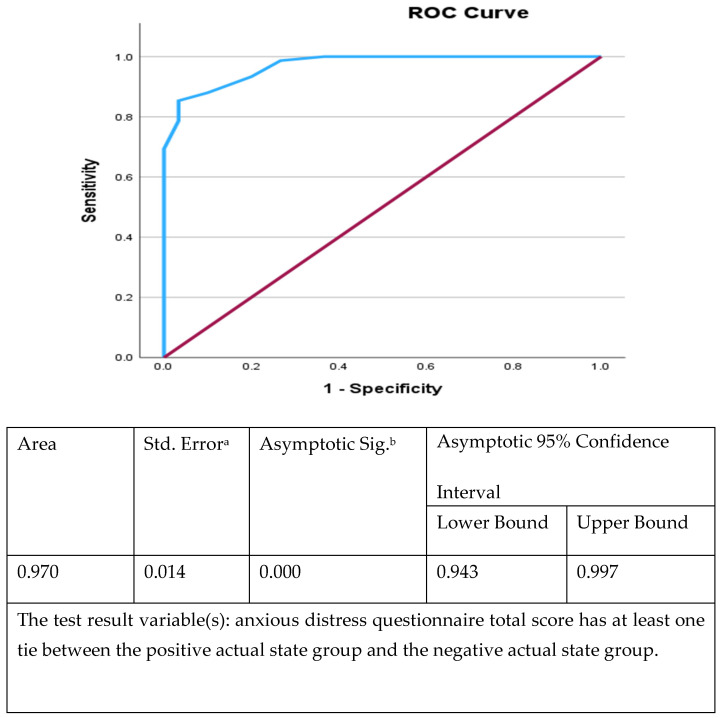
ROC curve illustrating the diagnostic performance of the ADS total score in identifying DSM-5-TR anxious distress. The area under the curve (AUC) was 0.97 (95% CI: 0.943–0.997), indicating outstanding discriminatory ability. ^a^ standard error of the AUC; ^b^ Asymptotic significance (*p*-value) of the AUC.

Receiver Operating Characteristic (ROC) curve analysis was performed to evaluate how effectively ADS differentiated individuals who met DSM-5-TR criteria for anxious distress from those who did not. As shown in Figure 1, the area under the curve (AUC) was 0.970 (standard error = 0.014, 95% CI: 0.943–0.997, *p* < 0.001), indicating outstanding diagnostic performance. According to the benchmarks proposed by Hosmer and Lemeshow (2000), an AUC between 0.90 and 1.00 reflects excellent diagnostic accuracy [17]. The ROC output also indicated at least one tied score between the positive and negative groups, a common occurrence in clinical datasets that does not materially affect the robustness of the AUC estimate.

### 3.4. Sensitivity, Specificity, Predictive Values, Likelihood Ratios

Table 4 presents sensitivity, specificity, corresponding 95% confidence intervals, and Youden Index values across candidate ADS cut-off scores. Although the highest Youden Index was observed at a cut-off score of 11, a threshold of ≥10 was retained as the optimal cut-off because it provided a clinically appropriate balance between sensitivity and specificity while minimizing false negatives in this tertiary-care psychiatric sample. Lower cut-off scores were associated with higher sensitivity but lower specificity, whereas higher cut-off scores increased specificity at the expense of sensitivity.

Table 5 presents the 2 × 2 contingency table comparing ADS classification at the selected cut-off score (≥10) with DSM-5-TR-based clinical diagnosis of anxious distress. Of the 75 participants who met DSM-5-TR criteria, 66 were correctly identified by the ADS (true positives), while 9 were misclassified as negative (false negatives). Among the 30 participants who did not meet clinical criteria, 27 were correctly classified as negative (true negatives), and 3 were incorrectly classified as positive (false positives).

As shown in Table 6, at the selected cut-off score of ≥10, the ADS demonstrated a sensitivity of 0.88 (95% CI: 0.78–0.94) and a specificity of 0.90 (95% CI: 0.73–0.98). The positive predictive value (PPV) was 0.96 (95% CI: 0.88–0.98), while the negative predictive value (NPV) was 0.75 (95% CI: 0.62–0.85). The positive likelihood ratio (LR+) was 8.80 (95% CI: 3.00–25.83) and the negative likelihood ratio (LR−) was 0.13 (95% CI: 0.07–0.25). These likelihood ratio values indicate strong diagnostic performance, suggesting that individuals with anxious distress were substantially more likely to obtain a positive ADS result than those without the condition, while a negative ADS result considerably reduces the probability of anxious distress. The relatively lower NPV compared with PPV is likely attributable to the high prevalence of anxious distress in the study sample (71%), as predictive values are influenced by condition prevalence. Overall, these findings support the clinical utility of the ADS as a screening instrument while indicating that a negative result does not fully exclude the presence of anxious distress.

### 3.5. Internal Reliability, Principal Component Analysis and Correlation Analysis

**Figure 2:**
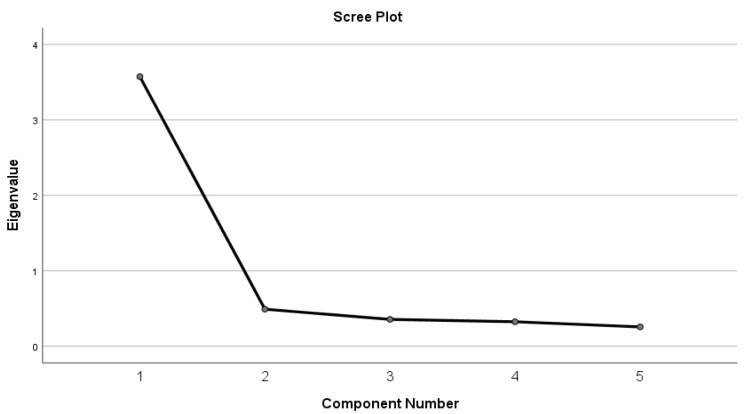
Scree plot from Principal Component Analysis (PCA) of the ADS items. A clear inflection after the first component supports a dominant single-component solution. Each dot represents the eigenvalue for a component, and the line connects these values to illustrate the scree pattern. A clear inflection after the first component supports a dominant single-component solution.

Cronbach’s alpha was calculated to assess the internal consistency of the ADS, yielding a coefficient of α = 0.897, indicating excellent internal reliability. To examine the dimensional structure of the scale, principal component analysis (PCA) was conducted. The Kaiser–Meyer–Olkin (KMO) measure of sampling adequacy was 0.879, indicating meritorious adequacy according to Kaiser’s criteria [18], and Bartlett’s test of sphericity was statistically significant (χ^2^(10) = 300.427, *p* < 0.001), confirming that the data were suitable for PCA(Table 7). The PCA identified a single dominant component with an eigenvalue of 3.57, accounting for 71.46% of the total variance (Table 8). Inspection of the scree plot showed a clear inflection after the first component, supporting a predominantly unidimensional component structure (Figure 2). Component loadings for all five items were high, ranging from 0.812 to 0.886, indicating strong contributions of each item to the shared dimension measured by the scale (Table 9). Corrected item–total correlations ranged from 0.707 to 0.808, indicating strong associations between individual items and the overall ADS score (Table 10). Deletion of any individual item did not improve Cronbach’s alpha, supporting retention of all five items. Collectively, these findings demonstrate strong internal coherence of the ADS and support a predominantly unidimensional component structure.

Spearman’s rank-order correlation analysis demonstrated a strong positive association between ADS scores and GAD-7 scores (*r* = 0.821, *p* < 0.01), supporting convergent validity with anxiety-related symptomatology (Table 11). A moderately strong positive correlation was also observed between ADS and MADRS scores (*r* = 0.677, *p* < 0.01), indicating that anxious distress was associated with greater depressive severity, although the MADRS represents a related but distinct construct. The correlation between GAD-7 and MADRS scores (*r* = 0.617, *p* < 0.01) further reflects the recognized overlap between anxiety and depressive symptom domains.

## 4. Discussion

The findings of this study indicate that anxious distress is highly prevalent among individuals with major depressive disorder (MDD). In the present sample, 71% of participants met DSM-5-TR criteria for the anxious distress specifier, which aligns closely with previous estimates suggesting that approximately 60–75% of individuals with MDD experience clinically significant anxiety symptoms during depressive episodes [19,20]. Consistent with prior literature, anxious distress in this study was associated with greater depressive severity [21]. Participants meeting criteria for the anxious distress specifier demonstrated higher levels of depressive symptomatology, reinforcing evidence that anxiety symptoms embedded within depressive episodes are linked to slower treatment response, increased relapse risk, and greater functional impairment [1,19,20,21].

Relative to existing assessment tools, the ADS offers several practical advantages. In contrast to longer clinician-administered or embedded instruments, the ADS is brief, self-administered, and specifically designed to operationalize DSM-5-TR anxious distress criteria. In the present study, the ADS demonstrated excellent internal consistency (Cronbach’s α = 0.897), a coherent unidimensional component structure, and outstanding diagnostic accuracy (AUC = 0.97).

Diagnostic performance indices further support the clinical utility of the ADS. A cut-off score of ≥10 provided an optimal balance between sensitivity (88%) and specificity (90%), supporting its potential use as an efficient screening threshold. Clinically, a positive ADS score at or above this cut-off strongly supports the presence of anxious distress and may prompt more detailed assessment or consideration of anxiety-informed treatment strategies. Conversely, a negative ADS score should be interpreted cautiously, particularly in high-prevalence psychiatric settings, and should not replace comprehensive clinical evaluation when clinical suspicion remains. Although the ADS demonstrated strong diagnostic accuracy at the selected cut-off, the negative predictive value indicates that some individuals with anxious distress may score below the threshold. Accordingly, a negative ADS result should not be interpreted as definitively excluding the presence of anxious distress. Instead, the ADS should be viewed as a screening and decision-support instrument rather than a substitute for clinical evaluation. When clinical suspicion persists despite a negative ADS result, further assessment through a comprehensive clinical interview may be warranted.

The high prevalence of anxious distress in this tertiary-care sample has important implications for interpreting predictive values. Because predictive values are influenced by condition prevalence, the relatively high prevalence observed in this study likely contributed to the high PPV. In settings with lower prevalence, such as primary care or community samples, the ADS may demonstrate comparatively higher negative predictive values but lower PPV. These considerations highlight the importance of interpreting ADS results within the clinical context and underscore the need for validation across populations with different baseline prevalence rates.

Importantly, ADS total scores provide a dimensional index of anxious distress severity rather than a direct operationalization of DSM-5-TR categorical severity levels. Whereas DSM-5-TR defines anxious distress severity based on symptom counts, the ADS captures symptom frequency and intensity on a continuous scale. Consequently, higher scores reflect both the number and frequency of anxious distress symptoms during the depressive episode. Scores approaching the empirically derived cut-off of ≥10 typically indicate endorsement of multiple symptoms occurring at least “sometimes” or “frequently,” which is broadly consistent with DSM-5-TR criteria requiring at least two symptoms present on the majority of days during the depressive episode. Scores near the cut-off threshold should therefore be interpreted with clinical judgment, particularly when symptom severity fluctuates or when contextual factors suggest possible anxious distress despite a borderline score. Accordingly, the ADS is intended to function as a screening and decision-support instrument to assist clinicians in identifying individuals who may meet criteria for anxious distress, rather than replacing comprehensive clinical assessment.

Construct validity was supported by strong correlations with established measures of anxiety and depression. The ADS demonstrated a strong association with the GAD-7 and a moderate-to-strong correlation with the MADRS, reflecting the well-documented overlap between anxiety and depressive symptom domains while indicating that the ADS captures anxiety-related symptoms within depressive episodes.

The development of the ADS also addresses an important gap in current clinical and research practice. Although the DSM-5-TR anxious distress specifier has clear prognostic implications, it is not operationalized within commonly used structured diagnostic interviews such as the SCID-5 or MINI. Consequently, identification of anxious distress often relies on unstructured clinical judgment guided by DSM-5-TR criteria, which may vary across clinicians and settings. The ADS was designed to provide a brief, standardized self-report instrument directly aligned with DSM-5-TR symptom definitions, facilitating more consistent identification of anxious distress in both clinical practice and research contexts.

Cultural factors may influence the presentation and reporting of anxious distress. In Malaysian populations, emotional distress may sometimes be expressed through somatic symptoms or generalized tension rather than explicit cognitive anxiety, and cultural norms surrounding emotional restraint or stigma may influence symptom disclosure [22,23]. These considerations highlight the importance of cross-cultural validation. Future studies should therefore examine the performance of the ADS across diverse cultural, linguistic, and healthcare settings.

Several limitations should be acknowledged. First, although DSM-5-TR specifies that anxious distress symptoms should occur on the majority of days during a depressive episode, it does not provide explicit guidance for operationalizing this criterion in self-report instruments. The ADS therefore used weekly frequency anchors as a pragmatic proxy for episode-level persistence, which may not fully capture symptom stability across prolonged episodes. In addition, because participants were asked to rate symptoms in relation to their current depressive episode rather than a fixed recall window, some degree of recall bias may occur, particularly among individuals experiencing longer episodes. This approach was chosen to maintain conceptual alignment with DSM-5-TR criteria, which define anxious distress in relation to the depressive episode rather than a specific timeframe. Future studies may explore strategies to improve temporal anchoring—for example, by clarifying the “recent period” of the current episode—while preserving diagnostic consistency with DSM-5-TR definitions.

Second, a separate standalone pilot study was not undertaken because the ADS was developed as a theory-driven instrument anchored directly to DSM-5-TR anxious distress criteria. Consistent with COSMIN recommendations for early instrument development, content validity was supported through expert review and embedded participant feedback evaluating item clarity and interpretability [9,24,25]. Nevertheless, future studies may benefit from additional pilot testing to further refine item wording and response anchors.

Third, diagnostic assessments were conducted by a single clinician, and formal inter-rater reliability was not evaluated. Test–retest reliability was also not assessed because anxious distress may fluctuate during active depressive episodes or treatment, and the assumption of temporal stability required for such analysis may not be met [26]. Future validation studies could address these limitations by recruiting larger samples and incorporating repeated ADS assessments over short intervals among clinically stable participants to evaluate temporal stability using intraclass correlation coefficients. In addition, inter-rater reliability of the DSM-5-TR diagnostic classification could be examined by having independent clinicians determine the presence of the anxious distress specifier and assessing agreement using Cohen’s kappa. Such studies would provide further evidence regarding the reliability and stability of ADS-based assessments.

Several additional psychometric considerations should also be noted. Internal consistency in the present study was evaluated using Cronbach’s alpha, which is widely used but assumes tau-equivalence among items and may not fully capture reliability when item contributions differ [27]. Future studies may therefore consider complementary reliability indices such as McDonald’s omega to provide additional estimates of internal consistency.

In addition, several measurement properties were not examined in the present study, including measurement error, responsiveness to treatment-related change, and formal discriminant validity. Because the available comparator instruments primarily assessed related constructs—namely anxiety symptoms (GAD-7) and depressive severity (MADRS)—the study focused primarily on convergent validity. Although anxious distress occurs within depressive episodes and is often associated with greater depression severity, future research incorporating instruments that assess theoretically distinct constructs or measures more directly evaluating anxious distress would allow a more comprehensive assessment of discriminant validity and the overall psychometric profile of the ADS.

Furthermore, the cross-sectional design of the present study limits evaluation of the scale’s responsiveness to therapeutic interventions. Longitudinal studies will therefore be important to determine whether ADS scores are sensitive to clinically meaningful treatment-related changes over time.

The DADSI was not routinely available within the clinical setting in which this study was conducted as it is a clinician-administered instrument that requires structured probing and trained raters. Similarly, the CUDOS-A is embedded within the broader CUDOS rather than functioning as a stand-alone anxious distress measure. For this initial validation study, the ADS was therefore evaluated against established measures assessing related constructs—namely the GAD-7 and MADRS—to examine convergent validity and diagnostic performance relative to DSM-5-TR clinical assessment. Nevertheless, the absence of direct comparison with other anxious distress–specific instruments represents a limitation of the present study. Future research could benchmark the ADS against instruments such as the DADSI and CUDOS-A within the same sample by administering these measures concurrently and comparing their diagnostic accuracy, agreement with DSM-5-TR clinical classification, and psychometric performance. Such comparative studies would help clarify the relative strengths, clinical feasibility, and incremental validity of the ADS in relation to existing assessment tools.

Additionally, although principal component analysis provided preliminary support for a unidimensional structure, confirmatory factor analysis (CFA) in larger independent samples will be required to further examine the structural validity of the scale and to evaluate measurement invariance across demographic and clinical subgroups. The present sample size also limited subgroup analyses examining potential effects of comorbid psychiatric conditions or demographic differences [28].

Another limitation is that the study sample consisted of English-speaking Malaysian adults with major depressive disorder recruited from a tertiary psychiatric center, with a predominance of female and Malay participants. These characteristics may limit the generalizability of the findings to other clinical populations or community settings. Future studies involving community samples, primary care settings, and other mood disorder populations such as bipolar disorder will be important to further evaluate the scale’s generalizability and measurement invariance across diverse populations. In addition, future research should consider formal translation and cross-cultural validation of the ADS in other languages and cultural contexts, following established guidelines for the cultural adaptation of patient-reported outcome measures. Such efforts would help evaluate the cross-cultural validity and broader applicability of the ADS across diverse populations and healthcare settings.

Finally, the duration of the current depressive episode was not systematically collected. Episode duration may influence the persistence and recall of anxious distress symptoms, and its absence limits the ability to examine whether symptom patterns differ across shorter versus more prolonged depressive episodes. Future studies should consider incorporating standardized assessment of episode duration to better characterize the clinical context in which ADS scores are obtained.

In summary, the ADS demonstrates promising psychometric properties as a brief self-report instrument for detecting anxious distress in individuals with major depressive disorder. With further validation in larger and more diverse samples, the ADS may provide a practical tool for supporting more consistent identification of anxious distress in both clinical practice and research settings.

## 5. Conclusions

The psychometric evaluation of the ADS indicates that the measure demonstrates strong performance, including excellent internal consistency, a coherent unidimensional structure, substantial convergent validity with established measures of anxiety and depression, and high diagnostic accuracy relative to clinical assessment guided by DSM-5-TR. An empirically derived cut-off score of ≥10 provided a clinically meaningful balance between sensitivity and specificity, supporting the ADS as a practical screening instrument for identifying clinically significant anxious distress within depressive episodes.

The ADS’s brief, self-administered format and direct alignment with DSM-5-TR anxious distress criteria address important limitations of existing clinician-administered or embedded measures. These characteristics support its feasibility for use in both routine clinical practice and research settings where efficient and standardized assessment of anxious distress is required.

The present findings should be interpreted in light of the study’s scope and sample characteristics. Validation was conducted in English-speaking Malaysian adults with major depressive disorder recruited from a tertiary psychiatric setting. Further validation is therefore required before extending the use of the ADS to other diagnostic groups, including bipolar disorder and persistent depressive disorder, as well as to culturally and linguistically diverse populations and community or primary-care contexts. In addition, longitudinal studies will be important to evaluate the temporal stability of the ADS, its responsiveness to treatment-related change, and its potential utility for monitoring symptom trajectories over time.

Overall, this study provides initial evidence that the ADS demonstrates promising psychometric properties within a tertiary-care MDD sample and may serve as a practical screening tool pending further external validation. By offering a concise instrument explicitly aligned with DSM-5-TR anxious distress criteria, the ADS contributes to efforts toward more standardized specifier-based assessment in mood disorders and provides a foundation for future cross-cultural, transdiagnostic, and longitudinal validation.

## Figures and Tables

**Table 1:** Mapping of ADS items to DSM-5-TR Anxious Distress Specifier.

DSM-5 TR Anxious Distress Criteria	ADS Item	ADS Questions
Feeling keyed up or tense	1	Have you been feeling nervous, tense or unable to relax most of the time?
Feeling unusually restless	2	Have you been feeling extremely restless that you were unable to sit still most of the time?
Difficulty concentrating because of worry	3	Have you been having difficulty concentrating due to worry or anxiety most of the time?
Fear that something awful might happen	4	Have you been worrying that something bad will happen most of the time?
Fear that one might lose control of oneself	5	Have you been worrying that you may lose control of yourself most of the time?

**Table 2:** Sociodemographic Characteristics of the Study Subjects (*N* = 105).

Variable	
Age, mean (sd)	33.10 (10.89)
Gender, *n* (%)	
Male	20 (19.0)
Female	85 (81.0)
Ethnic, *n* (%)	
Malay	70 (66.7)
Chinese	29 (27.6)
Indian	5 (4.8)
Others	1 (1.0)
Marital Status, *n* (%)	
Single	70 (66.7)
Married	29 (27.6)
Divorced	5 (4.8)
Widow	1 (1.0)
Education level, *n* (%)	
Primary	1 (1.0)
Secondary	27 (25.7)
Tertiary	66 (62.9)
Postgraduate	11 (10.5)
MDD (*n*, %)	
MDD only	93 (88.6)
MDD with ADHD	2 (1.9)
MDD with ASD	1 (1)
MDD with BPD	4 (3.8)
MDD with SUD	3 (2.9)
MDD with SAD	1 (1)
MDD with BDD	1 (1)
MDD severity (MADRS)	
Minimal/Absent	7 (6.7)
Mild	20 (19.0)
Moderate	43 (41.0)
Severe	35 (33.3)
Patient settings	
Inpatient	44 (41.9)
Outpatient	61 (58.1)

MDD = Major Depressive Disorder; ADHD = Attention-Deficit Hyperactivity Disorder; ASD = Autism Spectrum Disorder; BPD = Borderline Personality Disorder; SUD = Substance Use Disorder; SAD = Social Anxiety Disorder; BDD = Body Dysmorphic Disorder.

**Table 3:** Scoring of ADS.

Items	Mean	Std. Deviation
Item 1 (keyed up)	2.38	1.19
Item 2 (restlessness)	2.00	1.37
Item 3 (difficulty concentrating)	2.42	1.30
Item 4 (something awful might happen)	2.59	1.45
Item 5 (worrying about losing control)	2.42	1.47
Total score	11.94	5.69

**Table 4:** Diagnostic performance of the Anxious Distress Assessment Scale (ADS) across candidate cut-off scores.

ADS Cut-Off Score	Sensitivity (95% CI)	Specificity (95% CI)	Youden Index
≥8	0.99 (0.93–1.00)	0.73 (0.54–0.87)	0.72
≥9	0.93 (0.84–0.97)	0.80 (0.61–0.92)	0.73
≥10	0.88 (0.78–0.94)	0.90 (0.73–0.98)	0.78
≥11	0.85 (0.74–0.92)	0.97 (0.83–1.00)	0.82
≥12	0.79 (0.68–0.88)	0.97 (0.83–1.00)	0.76
≥13	0.69 (0.57–0.80)	1.00 (0.88–1.00)	0.69

Abbreviations: CI = confidence interval. Youden Index = Sensitivity + Specificity – 1. Confidence intervals were estimated using binomial methods.

**Table 5:** Contingency table comparing ADS classification with DSM-5-TR clinical diagnosis of anxious distress.

	Clinical Diagnosis−	Clinical Diagnosis+	Total
ADS− (≤10)	27 (TN)	9 (FN)	36
ADS+ (≥10)	3 (FP)	66 (TP)	69
Total	30	75	105

Abbreviations: ADS = Anxious Distress Assessment Scale; TN = true negative; FP = false positive; FN = false negative; TP = true positive.

**Table 6:** Diagnostic Accuracy of the ADS at the Selected Cut-off Score (≥10).

Parameter	Estimate	95% CI
Sensitivity	0.88	0.78–0.94
Specificity	0.90	0.73–0.98
PPV	0.96	0.88–0.98
NPV	0.75	0.62–0.85
LR+	8.80	3.00–25.83
LR−	0.13	0.07–0.25
Accuracy	0.89	0.81–0.94

Abbreviations: PPV = positive predictive value; NPV = negative predictive value; LR+ = positive likelihood ratio; LR− = negative likelihood ratio. Youden Index = Sensitivity + Specificity − 1. Confidence intervals were estimated using binomial methods.

**Table 7:** KMO and Bartlett’s Test.

Kaiser–Meyer–Olkin Measure of Sampling Adequacy.		0.879
Bartlett’s Test of Sphericity	Approx. Chi-Square	300.427
	Df	10
	Sig.	*p* < 0.001

**Table 8:** Total Variance Explained.

Component	Initial Eigenvalues			Extraction Sums of Squared Loadings		
	Total	% of Variance	Cumulative %	Total	% of Variance	Cumulative %
1	3.57	71.46	71.46	3.57	71.46	71.46
2	0.49	9.81	81.27			
3	0.36	7.11	88.39			
4	0.32	6.49	94.87			
5	0.26	5.13	100.00			

Extraction Method: Principal Component Analysis.

**Table 9:** Factor Loadings for the ADS.

	Component
	1
Item 1 (keyed up)	0.886
Item 2 (restlessness)	0.821
Item 3 (difficulty concentrating)	0.844
Item 4 (something awful might happen)	0.862
Item 5 (worrying about losing control)	0.812

Extraction Method: Principal Component Analysis. One component was retained based on eigenvalues > 1 and scree plot inspection.

**Table 10:** Corrected item–total correlations and Cronbach’s Alpha if Item Deleted for the Anxious Distress Assessment Scale (ADS).

	Scale Mean If Item Deleted	Scale Variance If Item Deleted	Corrected Item-Total Correlation	Cronbach’s Alpha If Item Deleted
Item 1	9.43	22.27	0.808	0.864
Item 2	9.81	21.73	0.712	0.882
Item 3	9.39	21.86	0.751	0.874
Item 4	9.22	20.52	0.773	0.869
Item 5	9.39	21.07	0.707	0.884

**Table 11:** Spearman Correlations between Anxious Distress Assessment Scale (ADS), MADRS and GAD-7.

	ADS	MADRS	GAD-7
ADS	1.000	0.677 **	0.821 **
MADRS	0.677 **	1.000	0.617 **
GAD-7	0.821 **	0.617 **	1.000

** *p* < 0.01 (two-tailed), MADRS = Montgomery–Åsberg Depression Rating Scale, GAD 7 = Generalized Anxiety Disorder-7.

## Data Availability

The datasets generated and analyzed during the current study are not publicly available due to patient confidentiality and institutional data protection regulations but are available from the corresponding author upon reasonable request and subject to ethical approval.
